# Molecular evolution of *Adh *and *LEAFY *and the phylogenetic utility of their introns in *Pyrus *(Rosaceae)

**DOI:** 10.1186/1471-2148-11-255

**Published:** 2011-09-14

**Authors:** Xiaoyan Zheng, Chunyun Hu, David Spooner, Jing Liu, Jiashu Cao, Yuanwen Teng

**Affiliations:** 1 Department of Horticulture, the State Agricultural Ministry Key Laboratory of Horticultural Plant Growth, Development and Quality Improvement, Zhejiang University, Hangzhou, Zhejiang 310058, China; 2 USDA, Agricultural Research Service, Department of Horticulture, University of Wisconsin, Madison, WI 53706-1590, USA

## Abstract

**Background:**

The genus *Pyrus *belongs to the tribe Pyreae (the former subfamily Maloideae) of the family Rosaceae, and includes one of the most important commercial fruit crops, pear. The phylogeny of *Pyrus *has not been definitively reconstructed. In our previous efforts, the internal transcribed spacer region (ITS) revealed a poorly resolved phylogeny due to non-concerted evolution of nrDNA arrays. Therefore, introns of low copy nuclear genes (LCNG) are explored here for improved resolution. However, paralogs and lineage sorting are still two challenges for applying LCNGs in phylogenetic studies, and at least two independent nuclear loci should be compared. In this work the second intron of *LEAFY *and the alcohol dehydrogenase gene (*Adh*) were selected to investigate their molecular evolution and phylogenetic utility.

**Results:**

DNA sequence analyses revealed a complex ortholog and paralog structure of *Adh *genes in *Pyrus *and *Malus*, the pears and apples. Comparisons between sequences from RT-PCR and genomic PCR indicate that some *Adh *homologs are putatively nonfunctional. A partial region of *Adh1 *was sequenced for 18 *Pyrus *species and three subparalogs representing *Adh1-1 *were identified. These led to poorly resolved phylogenies due to low sequence divergence and the inclusion of putative recombinants. For the second intron of *LEAFY*, multiple inparalogs were discovered for both *LFY1int2 *and *LFY2int2*. *LFY1int2 *is inadequate for phylogenetic analysis due to lineage sorting of two inparalogs. *LFY2int2-N*, however, showed a relatively high sequence divergence and led to the best-resolved phylogeny. This study documents the coexistence of outparalogs and inparalogs, and lineage sorting of these paralogs and orthologous copies. It reveals putative recombinants that can lead to incorrect phylogenetic inferences, and presents an improved phylogenetic resolution of *Pyrus *using *LFY2int2-N*.

**Conclusions:**

Our study represents the first phylogenetic analyses based on LCNGs in *Pyrus*. Ancient and recent duplications lead to a complex structure of *Adh *outparalogs and inparalogs in *Pyrus *and *Malus*, resulting in neofunctionalization, nonfunctionalization and possible subfunctionalization. Among all investigated orthologs, *LFY2int2-N *is the best nuclear marker for phylogenetic reconstruction of *Pyrus *due to suitable sequence divergence and the absence of lineage sorting.

## Background

The genus *Pyrus *L. belongs to the tribe Pyreae Baill. (the former subfamily Maloideae C. Weber) of the family Rosaceae [1] and is geographically divided into two groups: occidental pears and oriental pears [2]. The majority of oriental pears are native to China; a few are native to Japan and the Korean Peninsula. Chinese taxonomists agreed on 13 *Pyrus *species native to China, among which *P. betulaefolia *Bge. and *P. calleryana *Dcne. have retained characteristics believed to be ancestral for *Pyrus *[3] including the smallest fruit size and lowest carpel number. Based on morphological traits or crossing experiments, *P. × bretschneideri *Rehd., *P. × serrulata *Rehd., *P. × sinkiangensis *Yu, and *P. × hopeiensis *Yu are putative hybrids among other *Pyrus *species [4-6]. The circumscription of species, subspecies, and forms for occidental species remains controversial. It is believed that some cultivated pears frequently escaped from cultivation and became feral. These plants hybridize easily, both with cultivated and wild species, resulting in a number of intermediate forms and segregants [7]. Therefore, morphological characters are poor indicators of *Pyrus *phylogeny. Other data sources, like chemical characters, were used to distinguish some pear species [8], but these were plagued by low number of characters, polymorphisms, and environmental plasticity. During the last decade, molecular markers including RFLPs [9], RAPDs [10-12], genomic-SSRs [13,14], EST-SSRs [15,16] and AFLPs [10,17] have been applied in *Pyrus*. These data provided useful information on the origin of some cultivated pear groups, e.g. Chinese white pears (CWP), which are assigned to *P. × bretschneideri*. However, CWP are morphologically different from the so-called wild *P. × bretschneideri *in northern Hebei province [4]. They also show a close relationship to *P. pyrifolia *based on multiple molecular marker data and thus were treated as *P. pyrifolia *White Pear Group [11,14,17]. However, most of the studies have focused on the relationships of several oriental or occidental species or cultivar pear groups, and the phylogeny of the genus remains unresolved.

Plastid DNA sequence data and the internal transcribed spacer (ITS) of nuclear ribosomal DNA (nrDNA) have been used for plant phylogenetic reconstruction due to ease of amplification. In the Rosaceae, these data have been applied in phylogenetic studies at different taxonomic levels [18,19], but their utility is limited due to varied evolutionary rates [20-25]. Intra-individual ITS polymorphisms caused by incomplete concerted evolution of nrDNA arrays have been found in many Rosaceae [20,21,26,27]. Such polymorphisms provided evidence for hybrid origins of some species in *Rosa *[27], but they led to a poorly resolved phylogeny in *Malus *[21]. Similarly, our previous study in *Pyrus *[28] revealed a history of non-concerted evolution of ITS and a poorly resolved phylogenetic tree. Six non-coding regions of plastid DNA were found to be highly conserved in *Pyrus *[29], but they reflect only the maternal genealogies.

An alternative source of molecular sequence data, low-copy nuclear genes (LCNGs), has proven to be more phylogenetically informative than either ITS or plastid DNA [30]. These genes reflect biparental lineages and are less prone to homogenization [31-33]. Due to the accumulation of large number of gene sequences in GenBank, it is now possible to design taxa-specific primers. However, paralog and lineage sorting problems are still challenges to applying LCNGs to phylogenetic studies, since they may lead to topological incongruence similar to those caused by hybridization [31,34]. Gene duplication is a prominent feature of plant genome evolution, and duplicate segments account for 60% of the *Arabidopsis thaliana *genome [35]. In molecular phylogenetic studies, nuclear genes undergoing gene duplications or the birth-and-death process lead to problems in the identification of orthologs and paralogs and discordance between gene and species trees. Additionally, frequent gene duplications made the terms 'paralogy' and 'orthology' ambiguous. Thus the new terms 'inparalog' for paralogs that evolved after the ingroup speciation and 'outparalog' for those that evolved before ingroup speciation occurred [36]. Lineage sorting (or deep coalescence) results from random fixation of ancestral polymorphic alleles, which may induce similar topological incongruence to that of hybridization, and poses the most challenging problems for inter- and intra-specific phylogenetic inference [31,37]. However, lineage sorting is a random process, and fixation of ancestral alleles among species is rarely identical for two unlinked nuclear loci. Therefore, incongruence caused by hybridization and lineage sorting could be differentiated when comparing phylogenies based on multiple unlinked nuclear loci.

LCNGs that succeeded in other Rosaceae are potentially ideal nuclear markers for phylogenetic studies of *Pyrus*. The coding region of *GBSSI *has been successfully applied at intergeneric and higher levels [26,38], but the introns are too short and dispersed to be ideal gene regions for interspecific levels studies. Another gene region is the second intron of *LEAFY*, which is long enough and has been proven to be informative for studies at the interspecific level in Pyreae [39,40]. Complete coding sequences of two *LEAFY *loci have been isolated in *Pyrus *[41], and the corresponding genomic sequences in *Malus *species are available (DQ535885-*AFL1*, DQ535886-*AFL2*). Thus obtaining introns of *LEAFY *in the *Pyrus *taxa is possible.

Alcohol dehydrogenase gene (*Adh*) is one of the best-studied nuclear-encoded genes in plants. Two major ADH classes, class P with alcohol activity and class III with glutathione-dependent formaldehyde activity, have been identified in flowering dicot or monocot plants. The former is common for plants and the latter has been isolated in a few taxa including *Pisum sativum *(P80572) [42], *Oryza sativa *(U77637) and *Araobidopsis thaliana *(X82647) [43]. The *Adh *gene occurs in small gene families, and has proven to be a useful phylogenetic marker in the Poaceae and Paeoniaceae [44-46], but it is too complex a gene family or provided little phylogenetic resolution in other taxa such as the *Gossypium *and *Carex *[47,48]. Two distinct *Adh *loci (AF031900, AF031899) have been isolated in *P. communis *'Packham's Triumph' [49], thus it is possible to isolate *Adh *genes among *Pyrus *species. However, the only genomic *Adh *sequence from a species of Rosaceae is that from *Fragaria ananassa *(X15588) in GenBank [50], and the exon/intron structure is unknown in *Pyrus*. The phylogenetic utility of *Adh *coding regions and the intron region have not been determined in any Rosaceae taxa.

Since no LCNG analysis had been applied to phylogenetic studies of *Pyrus*, we explored the utility of *LEAFY *and *Adh*. In this study, a comparison of genomic and RT-PCR-based approaches yielded an initial description of the composition and functionality of the *Adh *gene family in *Pyrus*. The phylogenetic utility of *Adh *gene regions and the second intron of *LEAFY *were determined after examining the sequence divergence, gene duplications, lineage sorting and recombination.

*Malus *taxa were once assigned to *Pyrus*, but Miller treated *Malus *Mill. as a separate genus in 1768 due to graft incompatibility between the two [51]. *Malus *taxa originated before *Pyrus *taxa according to the fossil occurrence [52], and are here used as outgroups (Table 1).

**Table 1:** Plant taxa used in this study and subparalogs of *LFY1int2*, *LFY2int2 *and *Adh1-1 *recovered in each accession

Accessions ^a^	Species	Origin	Leaf source ^b^	Subparalogs or copy types^c^		
				
				*LFY1int2 *	*LFY*2int2	*Adh1-1*
**'Korlaxiangli'**	*P*. × *sinkiangensis *Yu	Xinjiang, China	CPGR	*a, b*	*N*	*a, b, c*
**'Cuiguan'**	*P. pyrifolia *	Cross	Zhejiang University	/	/	/
**'Nanguoli'**	*P. ussuriensis *	Liaoning Province, China	TU	*a*	*N, Ins8*	*a, b, c*
**'Flemish Beauty'**	*P. communis*	Belgium	ZZFI	/	/	/
**'Fuji'**	*M*. *domesitca *			/	/	/
'Chojuro'	*P. pyrifolia *Nakai	Kanagawa Pref. Japan	TU	*a*	*N*	*a, b*
'Nijisseiki'	*P. pyrifolia*	Chiba Pref. Japan	TU	*b*	*N*	*a, b, c*
'Yali'	*P. pyrifolia *White pear group	Hebei Province, China	TU	*a, b*	*Del2, S*	*a*
'Dangshansuli'	*P. pyrifolia *White pear group	Anhui Province, China	ZZFI	*a, b*	*N, Del2*	*a, b, c*
'Yaguang'	*P. ussuriensis *Maxim.	Liaoning Province, China	CPGR	*a*	*N, S*	*a, b*
'Jianbali'	*P. ussuriensis*	Liaoning Province, China	TU	*a*	*N, S*	*a, b, c*
*P. pashia *1	*P. pashia *D.Don	Yunnan Province, China	HRIYN	*b*	*N*	*a, c*
*P. pashia *2	*P. pashia*	Yunnan Province, China	HRIYN	*b*	*N*	*a, b, c*
*P. dimorphophylla*	*P. dimorphophylla *Makino	Mie Pref. Japan	TU	*a, b*	*N, S*	*a, b*
*P. calleryana*	*P. calleryana *Dcne.	South China	HRIYN	*a, b*	*N, Del2*	*a, c*
*P. fauriei*	*P. fauriei *Schneid.	Korea	TU	*a, b*	*S*	*a, b*
*P. betulaefolia*	*P. betulaefolia *Bge.	Gansu Province, China	CPGR	*b*	*N*	*a, c*
*P*. × *serrulata*	*P*. × *serrulata *Rehd.	Hubei Province, China	CPGR	*a, b*	*N*	*a, c*
*P. xerophila*	*P. xerophila *Yu	Gansu Province, China	GPI	*b*	*N*	*a, b*
*P*. × *hopeiensis*	*P*. × *hopeiensis *Yu	Hebei Province, China	Hebei Province, China	*a*	*N, Ins8*	*a, b*
*P*. × *phaeocarpa*	*P*. × *phaeocarpa *Rehd.	North China	CPGR	*a*	*N*	*a, b, c*
*P. hondoensis*	Nakai & Kikuchi	Middle Japan	TU	*a*	*N, Ins8*	*a, b, c*
*P. communis*	*P. communis *L.	Europe	TU	*b*	21-bp deletion	*a, b, c*
*P. elaeagrifolia*	*P. elaeagrifolia *Pall.	Turkey, Crimea, South East Europe	TU	*b, c*	*N*	*a, c*
*P. amygdaliformis*	*P. amygdaliformis *Vill.	Mediterranean area, South Europe	TU	*b, c*	*N*	*a, c*
*P. cossonii*	*P. cossonii *Rehder.	Algeria	TU	*b*	*N*	*a, b*
**outgroup**						
*M. sieboldii*	*M. sieboldii *(Regel.) Rehd	Yunnan Province, China	HRIYN	/	*N*	*a, b, c*
*M. rockii*	*M. rockii *Schneid.	Yunnan Province, China	HRIYN	/	/	*b, c*
*M. domestica *subsp. *chinensis*	*M. domestica *subsp. *chinensis *Li Y. N.-(Nai)	North China		/	/	*a, b, c*
*M. neidzwetzkyana*	*M. neidzwetzkyana *(Dieck) Langenf.	Xinjiang (Uygur Autonomous Region)		/	/	*b, c*
'Rall'	*M. domestica *Borkh			/	/	/

^a ^Accessions used for RT-PCR are in bold. ^b ^TU: Tottori University, Japan; CPGR: China Pear Germplasm Repository, Xingcheng, Liaoning Province; ZZFI: Zhengzhou Fruit Institute, Chinese Academy of Agriculture Science, Zhengzhou, Henan Province, China. GPI: Gansu Pomology Institute, Gansu Academy of Agricultural Science, Gansu Province, China; HRIYN: Horticultural Research Institute, Yunnan Academy of Agricultural Sciences, Kunming, Yunnan Province, China. ^c ^Different copy types of *LFY1 *and *LFY2 *identified by indels and phylogenetic analyses.

## Results

### Gene structure and paralog identification based on long Adh sequences

A total of 17 *Adh1 *and eight *Adh2 *long partial sequences were obtained by Genomic-PCR (G-PCR) using different primer sets (Table 2) in 'Cuiguan' (*P. pyrifolia*), 'Nanguoli' (*P. ussuriensis*), 'Korlaxiangli' (*P. sinkiangensis*), 'Flemish Beauty' (*P. communis*), *M. rockii*, *M. domestica *subsp. *chinensis *and 'Ralls' (*M. domestica*). All of these *Adh *genes encoded medium-chain ADH enzymes with 380 amino acid residues. After phylogenetic analyses, gene structure and sequence divergence comparisons, two paralogs representing *Adh1 *(*Adh1-1*, *Adh1-2*) and *Adh2 *(*Adh2-1*, *Adh2-2*) were identified.

**Table 2:** Primers used for PCR amplification and sequencing in this study

Target region		Primer sequence (5'-3')
*Adh*	Long partial region	Adh1-F1:ATGTCTAATACTGCTGGTCA Adh1-F2: TGATGTTTACTTCTGGGAGG Adh1-R1: GATTGAATTGTGTTCTTTA Adh1-R2: TGTGGATTATGCAACGAAGA Adh2-F: TGTTGACTTCTGGGATGCCAA Adh2-R: ATGCTAACGATGCACCGCAA
	Internal primer	Adh1-F5: AGGAGAATGCAAGGACTGCGCT Adh2-F5: CATTGCAAGTCTGAGGAAAG
	reduced *Adh1*	Adh1-F2 Adh1-R3: CAAAATGGTAGATAGGCTT
	Specific primers	spAdh1-F: TCTACCATTTTGTTGGGACT spAdh1-R: AACGCTTCCTGTACATTCAA
		spAdh2-F: GATTAATCACTTCCTCGGCA spAdh2-R: TAATATAGCCGGTGCACTCT
	
*LEAFY*	Long partial	LFY-F: TGTCGGAGGAGCCAGTGCAA LFY-R: GGCGTAGCAGTGCACATAGT
	*LFY1int2*	LFY1-F: TGGACGTTCATCAATAAAGA LFY1-R: AGTCGAACTAAATAGTTGAA
	*LFY2int2*	LFY2-F: GTGGGCCCATTTCCTGTAGT LFY2-R: GTTAAATCCGGTCAGATTAT
	*LFY2s*	LFY2S-F: CTGTATTGACTATTTCTGTC
	
		MLFY2-F: CGTACGCTTATTTCTACTGCA LFY2-R
	
*Actin *	Partial	Pact-F: CCATCCAGGCTGTTCTCTC Pact-R: GCAAGGTCCAGACGAAGG

**Figure 1:**
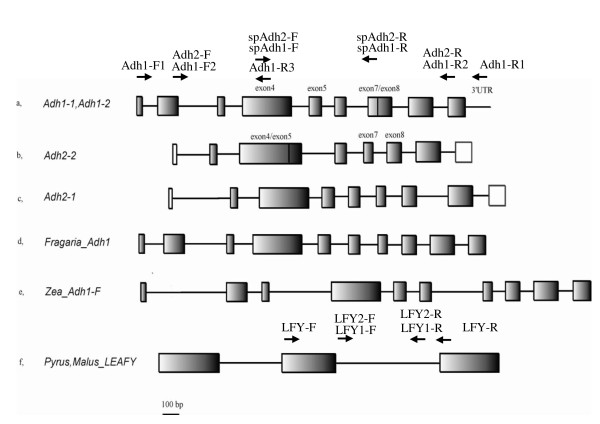
**Schematic diagram of *Adh *and *LEAFY *genes**. Open boxes represent exons, and connecting lines represent introns. Combinations of neighboring exons show the loss of intron 4 in *Adh2-2 *and the loss of intron 7 in *Adh1-1 *and *Adh1-2*. Arrows indicate the locations and directions of primers used for PCR amplification. Rows a, b and c are different *Adh *genes from *Malus *and *Pyrus *obtained in this study; Row d is the genomic *Adh *sequence from *Fragaria *(X15588); Row e is *Adh1-F *from *Zea *(AF050457); Row f is *LEAFY *from *Malus *and *Pyrus*. For Rows b and c, the exon 1 and intron 1of *Adh2-1 *and *Adh2-2 *were not amplified by primers Adh2-F and Adh2-R, and the empty boxes indicate incomplete exon 2 and exon 10.

As shown in Figure 1, *Adh2-1 *had a classical *Adh *gene structure with nine introns similar to *Zea mays*, *Fragaria ananassa*, and other characterized plant *Adh *genes [53]. Exon and intron codes described in this study were named following the classical gene structure to avoid confusion. *Adh2-2 *had lost intron 4, while both *Adh1-1 *and *Adh1-2 *had lost intron 7. *Adh1-1 *and *Adh1-2 *have the same gene structure, but the former was obtained by downstream primer Adh1-R1 located in the 3'UTR region, while the latter was obtained only by downstream Adh1-R2 located in the last exon (Figure 1). This indicates that the 3' UTR region of *Adh1-2 *may be divergent from that of *Adh1-1*. However, we were unable to obtain the 3'UTR region of *Adh1-2*. *Adh1-2 *obtained in 'Cuiguan' (*P. pyrifolia*) displayed a 20-bp deletion in exon 4. One of the three *Adh1-2 *clones in 'Korlaxiangli' (*P. sinkiangensis*) and one of the three *Adh1-2 *clones in 'Ralls' (*M. domestica*) displayed one or two 1-bp deletions, respectively, in exonic regions. A stop codon occurred in the exonic region in one of the two *Adh2-2 *sequences of *M. rockii*. These sequences were deemed putative pseudogenes and were removed from subsequent sequence analyses.

Among these four *Adh *paralogs (*Adh1-1*, *Adh1-2*, *Adh2-1 *and *Adh2-2*), only the intron regions of *Adh1-1 *and *Adh1-2 *could be aligned. Therefore, only the coding regions were used for nucleotide sequence divergence (NSD) and amino acid sequence divergence (ASD) comparisons. As shown in Table 3 NSD between *Adh1 *and *Adh2 *paralogs was as high as 0.3, while that within each paralog was lower than 0.02. NSD between *Adh2-1 *and *Adh2-2 *(0.19) was much greater than that between *Adh1-1 *and *Adh1-2 *(0.06). NSD of *Adh1-1 *and *Adh1-2 *between *Pyrus *and *Malus *were 0.027 and 0.035, respectively. All of these sequence divergence comparisons were consistent with the consequent phylogenetic inferences, indicating that our identification of paralogs was accurate. In most cases, NSD was greater than ASD between different homologs, while NSD was less than ASD within each homolog.

**Table 3:** Sequence divergence (mean value) of the coding regions between and within *Adh *homologs (excluding putative pseudogenes)

	*Pyrus Adh1-2 *(3)^c^		*Malus Adh1-2 *(1)		*Pyrus Adh1-1 *(6)		*Malus Adh1-1*(4)		*Malus Adh2-2 *(3)		*Pyrus Adh2-1 *(4)	
	**NSD^a ^**	**ASD^b ^**	**NSD**	**ASD**	**NSD**	**ASD**	**NSD**	**ASD**	**NSD**	**ASD**	**NSD**	**ASD**

*Pyrus Adh1-2*	**0.015^d ^**	**0.021**	**/**	**/**	**0.016**	**0.019**	**0.016**	**0.027**	**0.017**	**0.017**	**0.015**	**0.024**
*Malus Adh1-2*	0.035	0.027										
*Pyrus Adh1-1*	0.060	0.047	0.062	0.046								
*Malus Adh1-1*	0.058	0.050	0.062	0.050	0.027	0.039						
*Malus Adh2-2*	0.343	0.235	0.339	0.226	0.340	0.243	0.333	0.235				
*Pyrus Adh2-1*	0.322	0.185	0.318	0.191	0.321	0.191	0.318	0.190	0.197	0.159		

^a ^Sequence divergence based on nucleotide sequence (NSD)

^b ^Sequence divergence based on amino acid sequence (ASD)

^c ^The numbers in the parentheses indicate the number of sequences, and both NSD and ASD in the table represent mean values.

^d ^Sequence divergence within each homolog is highlighted in bold.

**Figure 2:**
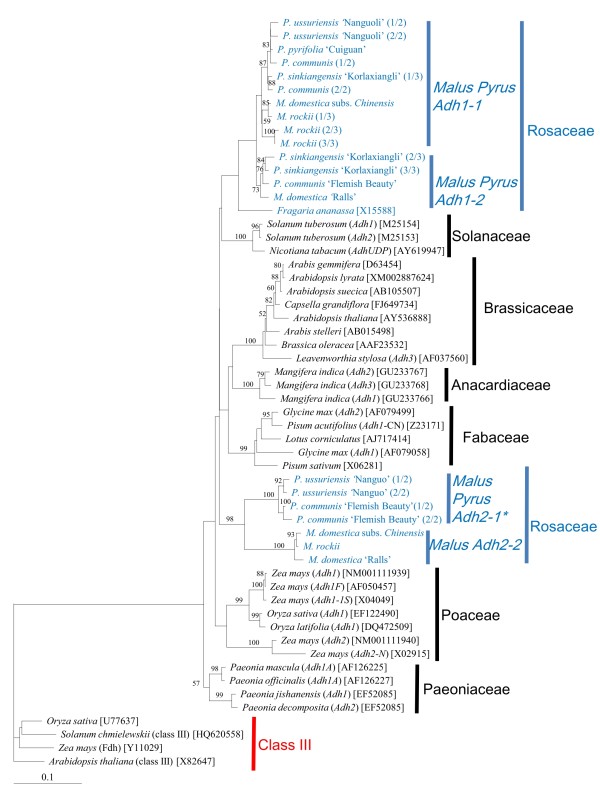
**Neighbor-joining (NJ) tree based on amino acid sequences of *Adh *loci from diverse plant taxa**. ADH sequences in Rosaceae are highlighted in blue. Numbers above the branches or near the branch nodes indicate bootstrap values (1000 replicates). GenBank accession numbers are in brackets. Multiple intra-individual clones for *Adh1 *(*Adh1-1 *and *Adh1-2*) and *Adh2 *(*Adh2-1 *and *Adh2-2*) are differentiated by the number in the parenthesis following the taxa name. *: Though *Adh2-1 *was not obtained by G-PCR in *Malus*, its transcription was detected by RT-PCR in 'Fuji' (*M. domestica*).

Both maximum parsimony (MP) (Additional file 1) and neighbor joining (NJ) (Figure 2) trees based on amino acid sequences of *Adh *genes from different plant taxa displayed similar topologies. Class III ADH formed clades separate from the putative class P ADH. The *Adh *genes from the Solanaceae, Brassicaceae, Anacardiaceae, and Fabaceae were monophyletic with high bootstrap values, suggesting that duplication events leading to these *Adh *genes occurred independently after diversification of these plant families. The *Adh *genes from Paeoniaceae and Poaceae also formed monophyletic clades; however, with low bootstrap values. Different *Adh *genes in *Malus *and *Pyrus *(Rosaceae) were not monophyletic. *Adh1-1 *and *Adh1-2 *in *Malus *and *Pyrus *formed two sister clades with *Fragaria ananassa *as their sister clade, suggesting that gene duplication leading to these two outparalogs occurred prior to diversification of *Malus *and *Pyrus*. *Adh2-1 *and *Adh2-2 *in *Malus *and *Pyrus *formed another clade. Due to lack of related sequences in other Rosaceae taxa, we cannot infer their origin. Among investigated families, Fabaceae is the most closely related to Rosaceae, but their *Adh *genes did not show a close relationship, indicating that the *Adh *genes have become highly diversified within each family.

### Phylogenetic analyses based on reduced Adh1 sequences

**Figure 3:**
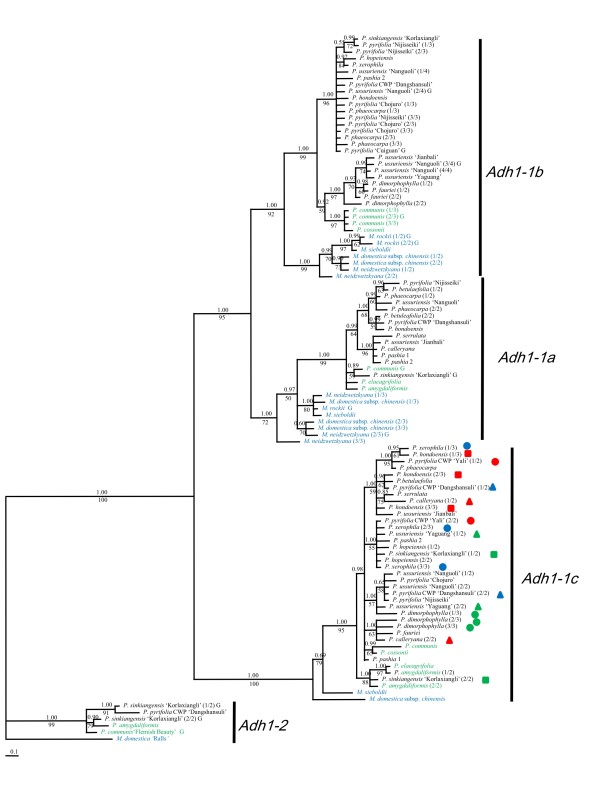
**Bayesian majority-rule consensus tree for reduced *Adh1***. Posterior probabilities and bootstrap values greater than 50 are provided above and below the branches, respectively. Outgroup accessions are highlighted in blue, while occidental species are in green. Multiple intra-individual clones for each of the three major clades (*Adh1-1a*, *Adh1-1b *and *Adh1-1c*) are differentiated by the fraction in the parenthesis following the taxon names, and those within the *Adh1-1a *clade are highlighted by different shapes and colors. The 'G' in the end of the taxa name indicates sequences obtained by G-PCR.

Only a short region containing intron 2 and intron 3 of *Adh1 *(reduced *Adh1*) were sequenced from more *Pyrus *species to investigate the phylogenetic utility of this region. *Adh1-1 *was preferentially amplified and only two *Adh1-2 *sequences were obtained in *P. amygaliformis *and 'Dangshansuli' (*P. pyrifolia*, CWP). Three subparalogs, *Adh1-1a*, *Adh1-1b*, *Adh1-1c*, were supported by the tree topologies (Figure 3). These were outparalogs that occurred before *Malus and Pyrus *diversification. Only *Adh1-1c *was obtained in all *Pyrus *accessions, while all of the three *Adh1-1 *outparalogs were derived in the same nine accessions. Multiple intra-individual polymorphic sequences representing one *Adh1-1 *outparalog could be recovered in some individuals. For example, five *Adh1-1c *sequences of *P. × hopeiensis *displaying autapomorphic mutations were monophyletic in the tree, but only one sequence was retained in the final dataset. Based on our previous experience, such small mutations were more probably caused by Taq polymerase errors during cloning and PCR. Among these subparalogs, the length of intron 2 varied from 228 to 262 bp while that of intron 3 varied from 114 to 117 bp, and subparalog associated indels were observed (data not shown). A string of thymine residues from 8 bp to 22 bp among *Adh1-1c *copies made alignment difficult, thus this region was removed from phylogenetic analyses. Two sequences possessing characteristics (indels and substitutions) of different *Adh1-1 *outparalogs were identified as recombinants by Recombination Detection Program (RDP) [54], and 13 more similar putative recombinants were identified manually by observing their conflicting positions within the alignment. These putative recombinants were probably artificial products created during PCR and were excluded from all analyses.

The final dataset contained 101 *Adh1-1 *and six *Adh1-2 *copies with an aligned length of 677 bp. The NSD within each *Adh1-1 *subparalog was very low, ranging from 0.011 to 0.013 (excluding *Malus *accessions) (Table 4). *Adh1-2 *from 'Ralls' (*M. domestica*) was selected as the outgroup for phylogenetic analyses. MP (data not shown) and Bayesian analyses (Figure 3) of this dataset resulted in similar phylogenetic trees with little difference in support values (the Bayesian posterior probabilities are generally higher than bootstrap percentage). *Adh1-1 *and *Adh1-2 *formed two separate clades. Within the *Adh1-1 *clade, *Adh1-1a *and *Adh1-1b *were closely related and formed a sister clade to *Adh1-1c*. *Adh1-1c *was obtained in all accessions, but the relationships were poorly resolved with extensive polytomies. The occidental species, *P. communis*, *P. amygdaliformis*, *P. elaeagrifolia *and *P. cossonii*, were not monophyletic. Most intra-individual polymorphic sequences were polyphyletic, e.g. *P. calleryana*, *P. xerophila*, 'Nijisseiki' (*P. pyrifolia*) and 'Korlaxiangli' (*P. sinkiangenesis*). *Adh1-a *and *Adh1-b *were only obtained in some accessions, thus the phylogenetic relationships in these two clades were incomplete, but the occidental species were monophyletic in both clades.

**Table 4:** Sequence variations of *Adh1-1 and LEAFY *subparalogs in *Pyrus *(excluding the *Malus *accessions)

	Reduced *Adh1*			The second intron of *LEAFY*	
		
	*Adh1-1a*	*Adh1-1b *	*Adh1-1c*	*LFY1int2*	*LFY2int2 *
N ^a^	37	30	17	53	43
NSD ^b^	0.012	0.013	0.011	0.019 (0.016)^d^	0.029 (0.028)^e^
PI ^c^	25/617	21/633	11/645	61/653 (42/653)^d^	46/562 (40/553)^e^

^a ^The number (N) of sequences used for analyses.

^b ^Mean sequence divergence of nucleotide sequence (NSD) calculated by the K-2P method.

^c ^The number of parsimony-informative (PI) sites after alignment.

^d ^PI sites and NSD in brackets were calculated after excluding *LFY1int2-c*.

^e ^PI sites and NSD in brackets were calculated after excluding *LFY2int2-Ins8 *and *LFY2int2-Del2*.

### Transcription of Adh homologs

**Figure 4:**
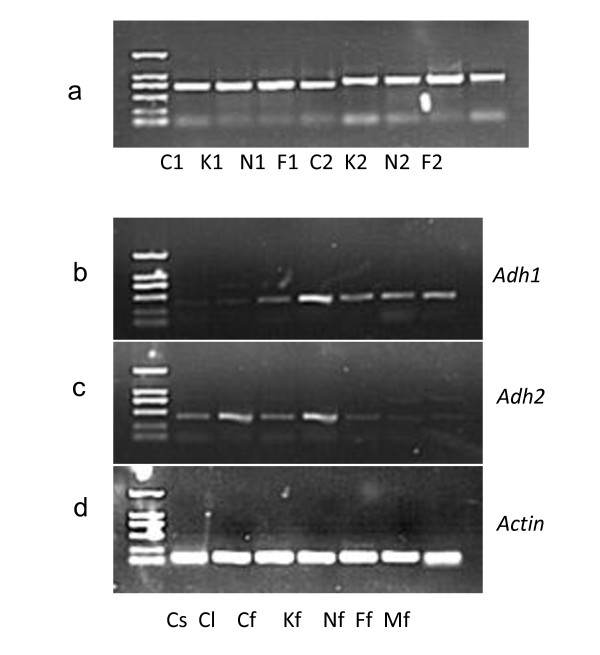
**Genomic (SG)-PCR and RT-PCR of *Adh1 *and *Adh2***. Bands of the molecular marker from the bottom up denote size standards of 0.1 kb, 0.2 kb, 0.5 kb, 0.75 kb, 1 kb and 2 kb. Row a is SG-PCR of *Adh1 *and *Adh2 *in 'Cuiguan'(C1, C2), 'Korlaxiangli' (K1, K2), 'Nanguoli' (N1, N2) and 'Flemish Beauty'(F1, F2); Rows b, c and d are RT-PCR of *Adh1*, *Adh2 *and control *Actin *gene in investigated tissues and cultivars. Cs, Cl and Cf denote seed, leaf and fruit of 'Cuiguan', respectively; Kf, Nf, Ff and Mf-fruit denote fruit of 'Korlaxiangli', 'Nanguoli', 'Flemish beauty' and 'Fuji', respectively.

Specific genomic PCR (SG-PCR) and RT-PCR using locus specific primers produced expected bands in the genomic DNA and cDNA samples. Both *Adh1 *and *Adh2 *were transcribed in all investigated tissues and cultivars (Figure 4). To investigate the transcription of different *Adh *homologs, phylogenetic analyses including sequences derived from SG-PCR, G-PCR and RT-PCR were conducted for *Adh1 *and *Adh2 *separately, and the putative pseudogenes identified above were also included to enhance the findings on functionality of *Adh *homologs.

As indicated in Additional file 2 the transcription of an *Adh1-2 *copy was not observed. We speculate that *Adh1-2 *is a nonfunctional outparalog, since putative pseudogenes have been identified and its 3'UTR region is divergent from *Adh1-1 *as described above. Among the *Adh1-1 *outparalogs the most frequently cloned *Adh1-1c *was not recovered by RT-PCR, indicating that it was a degenerate outparalog. This also explains its preferential amplification. Transcription of the other *Adh1-1 *outparalogs was detected among different tissues or cultivars. Transcription of *Adh2-1 *could be detected in all investigated tissues and cultivars. An *Adh2-1 *sequence was not obtained in the three *Malus *accessions by G-PCR, but its transcription was found in 'Fuji' (*M. domestica*) (Additional file 3). *Adh2-2 *was only observed in *Malus *taxa, and transcription of this paralog was not detected. We could not deduce distinct tissue-specific or cultivar-specific expression for either *Adh1-1 *or *Adh2-1*. Several anomalous *Adh2-1 *copies with only intron 6 were recovered by cloning of RT-PCR products in 'Flemish Beauty' (*P. communis*) and 'Nanguoli' (*P. ussuriensis*) (HQ912054, HQ912055 and HQ912056). Since intron-containing cDNA has not been reported, those copies may be amplified due to genomic DNA contamination and indicate existence of an additional *Adh2 *paralog with more intron loss in *Pyrus*.

### Sequence variation and paralogs of LEAFY

Twenty-six *LFY1 *and 27 *LFY2 *sequences including a partial exon 2 were obtained. Four groups with different length variations in the exon region were observed: *LFY1-Malus *(407 bp), *LFY1-Pyrus *(395 bp), *LFY2-Malus *(410 bp) and *LFY2-Pyrus *(401 bp), which were congruent with those from RT-PCR in *P. pyrifolia *'Housui' (*LFY1*-AB162029, *LFY2*-AB162035) and *M. domestica *'Fuji' (*LFY1*-AB162028 *LFY2*-AB162034), respectively. The length of these indels only had an effect on the length of the deduced amino acid sequences. Among *Pyrus *accessions, NSD of this partial exon 2 between *LFY1 *and *LFY2 *was relatively high (0.076), while that within *LFY1 *and *LFY2 *was low at 0.013 and 0.015, respectively (data not shown).

The entire intron 2 of *LFY1 *among *Pyrus *species ranged from 774-783 bp, while that of *LFY2 *ranged from 670 to 700 bp. *LFY1int2 *and *LFY2int2 *were amplified in all accessions using specific primers, but *LFY2int2 *of *M. rockii *and *M. domestica *subsp. *chinensis *were amplified by another forward primer MLFY2-F (Table 2) due to a large deletion (approximately 220 bp, GU991522 vs DQ535886) in these two accessions. *LFY2int2 *of all *Malus *accessions contained a 211-bp insertion, which made alignment difficult and was removed from the analyses. NSD within *LFY1int2 *and *LFY2int2 *among *Pyrus *accessions was 0.019 and 0.029, respectively (Table 4), while that between *Malus *and *Pyrus *was much higher at 0.057 for *LFY1int2 *and 0.066 for *LFY2int2 *(data not shown).

Sequence variation and phylogenetic analyses of *LFY1int2 *suggest two subparalogs, *LFY1int2-a *and *LFY1int2-b*, among *Pyrus *species. Compared with *LFY1int2-a*, *LFY1int2-b *contains a 6-bp insertion. Recovery of *LFY1int2-a *and *LFY1int2-b *in each accession is shown in Table 1. Among oriental species, NSD within *LFY1int2-a *was lower than that within *LFY1int2-b *for both ORF regions (0.002, 0.009) and intron regions (0.005, 0.013). Between *LFY1int2-a *and *LFY1int2-b*, NSD was 0.007 for the ORF region and 0.017 in for the intron region. For *LFY2int2*, subparalogs with an 8-bp insertion (*LFY2int2-Ins8*) and 2-bp deletion (*LFY2int2-Del2*) were only recovered in a few accessions. The common *LFY2int2-N*, with no indel, was recovered in all accessions but 'Yali' (*P. pyrifolia*, CWP) (Table 1). Coexistence of these subparalogs in one individual could be detected by direct sequencing due to the fixed position of indels. A minimum of three clones were sequenced. It was found that PCR or direct sequencing sometimes did not reflect the subparalogs existing in one genome, probably due to amplification preference of different nuclear alleles. For example, direct sequencing of *LFY2int2 *in *P. calleryana *identified *LFY2int2-N*, but a *LFY2int2-Del2 *sequence was obtained by cloning.

Several anomalous *LFY2int2 *copies were exclusively found in particular accessions. *LFY2int2 *from *P. communis *had a 21-bp deletion. *LFY2int2 *from *P. fauriei *had a 525-bp insertion that was partially homologous (reverse and complement) to the noncoding region of the S-RNase gene (AB308360), and was named *LFY2int2-S*. To eliminate the possibility of genetic recombination during PCR, all accessions were tested with an insert-specific upstream forward primer 'LFY2S-F' (Table 1) and the reverse primer LFY2-R. As a result, the *LFY2int2-S *was detected in 'Yali' (*P. pyrifolia*, CWP), 'Jianbali' (*P. pyrifolia *CWP), and 'Yaguang' (*P. ussuriensis*), but the sequences were not included in the phylogenetic analyses. *LFY2int2-S *in *P. fauriei *was still included in the dataset after the exclusion of its large insertion.

### Phylogenetic analyses of LFY1int2 and LFY2int2

*Malus sieboldii *and *M. domestica *(DQ535885-ALF1, DQ535886-ALF2) without the 220-bp deletion in *LFY2int2 *were used as outgroups in phylogenetic analyses of *LFY1int2 *and *LFY2int2*. Putative recombinants were identified by RDP or by investigating abnormal substitution patterns and ambiguous alignment positions. The putative recombinants displayed unique substitutions of sequences from two distinct subclades and always formed well-separated clades in the tree, thus they were excluded from the final analyses (data not shown). A total of 57 *LFY1int2 *(four from *Malus*) and 46 *LFY2int2 *(three from *Malus*) sequences were included in two separate datasets. Excluding sequences from *Malus*, the *LFY1int2 *dataset had an aligned length of 653 sites, of which 61 (9.3%) were parsimony informative, while *LFY2int2 *had an aligned length of 562 sites and 46 (8.2%) were parsimony informative (Table 4). Similarly, only Bayesian trees were shown for both *LFY1int2 *and *LFY2int2 *datasets.

**Figure 5:**
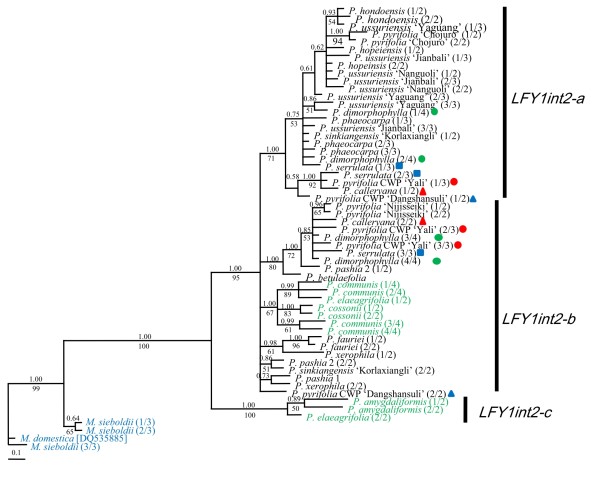
**Bayesian majority-rule consensus trees for *LFY1int2***. Posterior probabilities and bootstrap values greater than 50 are provided above and below the branches, respectively. Outgroup accessions are highlighted in blue, while occidental species are in green. Multiple intra-individual clones are differentiated by the fraction in the parenthesis following the taxon names. Accessions possessing both *LFY1int2-a *and *LFY1int2-b *are marked by different shapes and colors.

As shown in Figure 5, *LFY1int2-a *formed a monophyletic clade. The relationships within this clade were largely unresolved; however, close relationships among *P*. × *serrulata*, *P. calleryana *and 'Yali' (*P. pyrifolia*, CWP) were resolved with high bootstrap support. Most of the subclades of *LFY1int2-b *were unresolved polytomies sister to *LFY1int2-a*. The occidental species were not monophyletic, since three sequences from *P. amygdaliformis *and *P. elaeagrifolia *formed a highly supported independent clade. These three sequences, representing a paralog termed *LFY1int2-c*, displayed many unique variations and shared several substitutions with outgroup accessions. *LFY1int2-c *was reamplified in these four occidental species, and could be frequently cloned in *P. amygdaliformis *and *P. elaeagrifolia*. The origin of *LFY1int2-c *is unclear.

**Figure 6:**
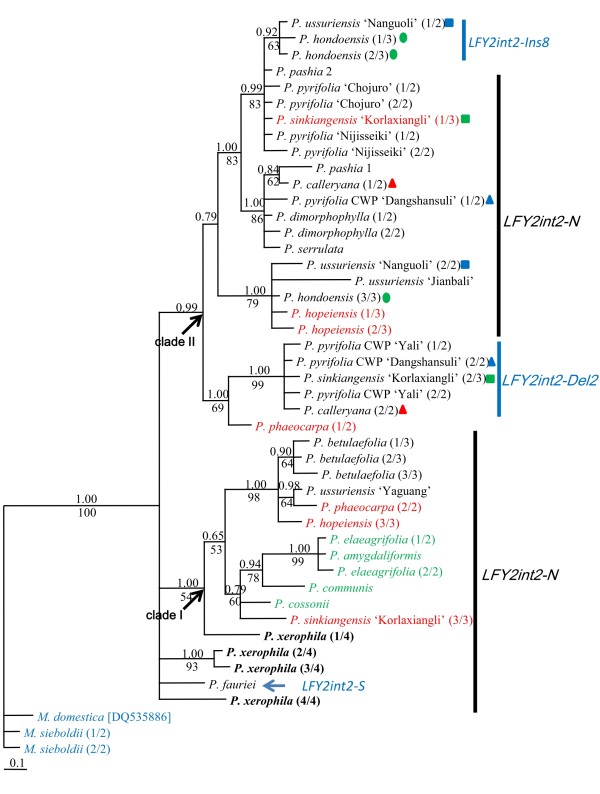
**Bayesian majority-rule consensus trees for *LFY2int2***. Posterior probabilities and bootstrap values greater than 50 are provided above and below the branches, respectively. Outgroup accessions are highlighted in blue, and occidental species in green. Multiple intra-individual sequences are differentiated by the fraction in the parenthesis following the taxon names. Accessions possessing both *LFY2int2-N *and *LFY1int2-Del2 *or *LFY1int2-Ins8 *are marked by different shapes and colors. Excluding the two inparalogs of *LFY1int2-Del2 *or *LFY1int2-Ins8*, intra-individual sequences representing *LFY2int2-N *are highlighted in red.

Relationships inferred by *LFY2int2 *were much better resolved, with higher support values and fewer polytomies (Figure 6), than those of *LFY1int2*., but the relationships within most subclades were still unresolved. Two major clades were resolved. In clade I, the four occidental species and a clone of 'Korlaxiangli' (*P. sinkiangensis*) formed a subclade sister to that of oriental pear including 'Yaguang' (*P. ussuriensis*), *P. betulaefolia*, *P. × phaeocarpa *and *P. × hopeiensis*. In clade II *LFY2int2-Ins8 *and *LFY2int2-Del2 *were monophyletic and mixed with *LFY2int2-N *sequences, thus they were inparalogs of recent origin. It is notable that the four *LFY2int2-N *sequences from *P. xerophila *were putative recombinants. They shared mutations with sequence from multiple subclades and also had unique mutations. Phylogenetic positions of these *P. xerophila *sequences were unresolved: *P. xerophila *(2/4) and *P. xerophila *(3/4) were sisters and formed a separate clade, *P. xerophila *(4/4) also formed a separate clade, and *P. xerophila *(1/4) shared mutations with occidental species and formed a separate subclade within clade I. It seems impossible that divergent copies in one genome are all recombinants, thus these sequences were included in the phylogenetic analyses and are highlighted in bold in Figure 6. Similarly, the *LFY2int2-S *of *P. fauriei *formed an unresolved separate clade.

## Discussion

### Frequency of Adh and LEAFY duplication

Gene duplication plays an important role in increasing the diversity of gene function and expression, which can enable plants to colonize diverse habitats. Most monocots and dicots have at least two *Adh *genes, indicating that an initial *Adh *gene duplication occurred before the divergence of these plant taxa, and separate duplications have subsequently taken place [46]. Two major loci in *Malus *and *Pyrus*, *Adh1 *and *Adh2*, are outparalogs derived from an ancient gene split. Based on an estimated 0.66% rate of nucleotide substitution per million year of *Adh *in *Drosophila *[55] and 0.2-0.3% in mammalian nuclear genes [56], the split occurred approximately 50 million years ago. In maize *Adh1 and Adh2 *share 87% identity at the amino acid sequence level but are located on different chromosomes and differ in the level of tissue-specific expression [57]. The expression of three *Adh *genes with 85% and 87% shared amino acid identity in *Vitis vinifera *varied in developmental stage of grape berries and affinity to either ethanol or acetaldehyde as a substrate [58]. ADH from apple had optimal acetaldehyde activity at pH 5.5-6.0 and ethanol activity at pH 7.0-10.0 [59]. Therefore, *Adh1-1 *and *Adh2-1 *in *Pyrus *(ASD = 0.170, Table 3) with less shared identity, likely also have diversified their expression patterns and substrate affinity. ADH plays an essential role in the biosynthetic pathway of aroma volatiles in apple and pear fruits by reducing aldehydes to alcohols [60-62]. It will be interesting to determine functional divergence of *Adh *genes and kinetic properties of the corresponding ADH enzymes in *Pyrus *and *Malus*.

Two paralogs representing *Adh1 *(*Adh1-1 *and *Adh1-2*) and *Adh2 *(*Adh2-1 *and *Adh2-2*) were observed by G-PCR. Among these the *Adh2-1 *may be the most ancestral since it has the classical nine-intron *Adh *structure, which is widely conserved among angiosperms and gymnosperms [53]. Intron losses have been found in *Adh *genes of diverse taxa like *Arabidopsis thaliana *[63,64] and *Mangifera indica *[65]. In some species of *Leavenwortia*, an expressed intronless *Adh3 *locus occurs and is thought to have arisen by an mRNA intermediate [66]. The single intron loss found in our study lends support to the 'intron exclusion hypothesis', which suggests that a single intron could be precisely removed by double strand breaks from a multiple-intron gene [67]. *Adh1-2*, with the same gene structure as *Adh1-1*, was a putative nonfunctional outparalog derived by gene duplication (loss of intron 7, Figure 1). Gene duplication leading to *Adh1-2*, as inferred by the tree topology (Figure 2), occurred before *Malus *and *Pyrus *diverged, but probably after diversification of Pyreae taxa, since the orthologous *Adh *gene in *Fragaria *(Rosoideae) displayed the classical nine-intron structure. Additionally, the NSD between *Adh1-1 *and *Adh1-2 *in *Pyrus *was 0.06 (Table 3), which was much lower than that between *Adh1-1 *in *Pyrus *and *Adh *in *Fragaria *(0.10) (data not shown). However, the NSD in the coding region between these outparalogs was too low to confirm the non-functionality of *Adh1-2*. *Adh2-1 *was not obtained in *Malus *accessions by G-PCR, probably due to an amplification preference for *Adh2-2*, but its transcription was detected in 'Fuji' (Additional file 3). *Adh2-2 *was recovered neither in *Pyrus *by G-PCR and SG-PCR nor in *Malus *or *Pyrus *by RT-PCR. Compared with *Adh1-1 *and *Adh1-2*, the ASD between *Adh2-1 *and *Adh2-2 *was much higher (Table 3) and exon/intron structure also varied (Figure 1). Due to the lack of highly homologous *Adh2 *sequences from other Rosaceae taxa, is the origin of *Adh2-2 *is uncertain. It may be a duplicated inparalog derived from *Adh2-1 *and restricted to *Malus *taxa or a functional outparalog that appeared before *Malus *and *Pyrus *diverged and was subsequently lost during diversification of *Pyrus *species. The latter theory is similar to the paralog sorting of *RPB1 *and *RPB2 *in different core eudicots taxa [68]. Specific RT-PCR for additional tissues and specific G-PCR for more Rosaceae taxa will be needed to examine the origin and transcription of *Adh2-2*. Three subparalogs representing *Adh1-1 *(*Adh1-1a*, *Adh1-1b*, *Adh1-1c*) were identified by accession sequencing (Figure 3), although *Adh1-1c *may not be transcribed. It is unknown whether similar subparalogs have evolved for *Adh1-2 *and *Adh2-2*.

Our study revealed that ancient and recent duplications led to the complex structure of *Adh *outparalogs in *Pyrus *and *Malus *resulting in neofunctionalization, nonfunctionalization and possible subfunctionalization, the three common fates of gene duplications. *Adh *homologs in *Malus *and *Pyrus *were more complex than those in other angiosperms like *Paeonia *[69], grasses [44] or legumes [70], but similar to those in *Gossypium *[48]. *Gossypium *has at least seven *Adh *loci of two primary lineages in diploid species and the *Adh *gene family is dynamic with pseudogenization and gene elimination. Genomic data suggest that almost all angiosperms, perhaps even all plant groups, have experienced one to several rounds of polyploidy [71,72]. Though *Malus *and *Pyrus *accessions used in this study were all diploid (x = 17), Pyreae taxa with x = 17 are derived from autopolyploidization of the formerly Spiraeoid ancestors with x = 9 [30]. This apparently accounts for such complex paralogs, and similar *Adh *gene structures could be imputed for other Pyreae taxa with x = 17.

*LEAFY *was first found to be a homeotic gene encoding a transcription regulator for differentiation of the floral meristem and flowering time in *Arabidopsis *and was expected to be a single-copy gene in diploid angiosperms [73]. In our study, two major lineages, *LFY1 *and *LFY2*, were recovered in both *Pyrus *and *Malus*, as in many other Pyreae taxa including the formerly Spiradeae taxa with x = 9 [39,41], suggesting gene duplication of these two paralogs before diversification of the Rosaceae. *LFY1 *and *LFY2 *in apple are located on distinct chromosomes and thus are not alleles [74]. In a study including the pear cultivars 'Housui' (*P. pyrifolia*) and 'Barlett' (*P. communis*), the transcriptional patterns of two *LEAFY *homologs differed in developmental stages and tissues, and each homolog varied among plant taxa [41]. In our study, genus-specific and locus-specific indels were discovered in coding regions, which would alter the length of the corresponding amino acid sequences. These might be responsible for the diversification of *LEAFY *gene functions.

Multiple inparalogs of *LFY1int2 *and *LFY2int2 *were observed, and their recovery varied among our accessions (Table 1). *LFY1int2-a *and *LFY1int2-b *were unequally observed among *Pyrus *species (Table 1). *LFY1int2-a *is monophyletic with shorter branch lengths than *LFY1int2-b *(Figure 5), suggesting it might be an inparalog derived from *LFY1int2-b *by a recent duplication. This would have occurred after the divergence of occidental and oriental pears, because only *LFY1int2-a *is not found in occidental pears. Consequently, *LFY1int2-b *was lost during diversification of some oriental species, which explains paralog sorting during diversification of *Pyrus*. Three *LFY1int2-c *sequences in two west Asian species (*P. amygdaliformis *and *P. elaeagrifolia*) formed a separate clade (Figure 5). We suggest two possible explanations for their origin: 1) they are pseudogenes derived from *LFY1int2-b*, but have evolved more rapidly, and thus are highly divergent from *LFY2int2-b*; 2) they represent another outparalog of *LFY1int2*, derived from gene duplication that occurred before diversification of the occidental species, that was subsequently lost in some occidental species. However, only the second intron of *LFY1int2-c *was sequenced. To help differentiate between these two possibilities the entire exonic region must be obtained and its presence in more *Pyrus *species investigated. For *LFY2int2*, the common *LFY2int2-N *was recovered in all accessions but 'Yali' (*P. pyrifolia*, CWP). *LFY2int2-Ins8 *and *LFY2int2-Del2 *are inparalogs that originated recently after *Pyrus *diversification and were only recovered in a few accessions (Figure 6). *LFY2int2-S*, with a long insertion homologous noncoding region of S-RNase gene, was similar to functional *AFL1a *copies found in some apple cultivars [74]. Genomic Southern analysis also showed that apple had other homologues in addition to *AFL1 *and *AFL2 *[75]. However, the relationships among these homologs have not been published. Only a few accessions contained *LFY2int2-S *(Table 1), and only the one from *P. fauriei *was included in our analyses. It is unknown how the intron of the RNase gene was inserted in the second intron of *LFY2 *and whether *LFY2int2-S *is functional. Additional research found both *LFY2int2-S *and *LFY2int2-N *in multiple species, and showed the *LFY2int2-S *sequences were all highly divergent from *LFY2int2-N *even after exclusion of the large insertion (unpublished data).

### Incongruence and poor resolution

Paralogs and lineage sorting are two major challenges when conducting phylogenetic analyses based on LCNGs, because they can lead to incongruent patterns similar to those resulting from hybridization and polyploidization [31]. Paralogs reflect a horizontal event, the gene duplication in one species, while orthologs reflect a vertical event, the speciation in a lineage [36,76]. Thus, it is crucial to differentiate paralogs from orthologs by investigating their origins and monophyletic positions in the tree. We have clearly identified the outparalogs and inparalogs for both *Adh *and *LEAFY *genes. If inparalogs representing *LFY1int2 *and *LFY2int2 *were not identified, *P. calleryana *possessing both *LFY1int2-a *and *LFY1int2-b *as well as *LFY2int2-N *and *LFY2int2-Del2 *would be polyphyletic in both gene trees (Figure 5 and 6) and presumed to be hybrids involving other *Pyrus *species. However, *P. calleryana *is one of the most ancestral species in *Pyrus *and should not be a hybrid of other *Pyrus *species. Additionally, *Pyrus ussuriensis*, *P. × hopeiensis*, *P. × phaeocarpa *and *P. hondoensis *containing only *LFY1int2-a *fell in the same clade. This shows a close relationship (Figure 5). However, *P. betuleafolia *was not in the clade and only contained *LFY1int2-b*. These findings were inconsistent with the hypothesis that *P. betuleafolia *was involved in the origin of *P. × hopeiensis *and *P. × phaeocarpa *and to the phylogeny based on *LFY2int2-N*.

Interspecific hybridization has been considered the major mode of evolution for *Pyrus *[52], and LCNG has been useful for testing the hypothesis of hybridization, since homologs of a nuclear locus from both parents could be detected in putative hybrids through cloning [31]. Excluding the possibility of paralogs, incongruence caused by hybridization and lineage sorting could be differentiated by comparing phylogenies of multiple unlinked nuclear loci. Only *Adh1-1c *and *LFY2int2-N *are shown to be two independent orthologs, and they were recovered in all accessions (except *LFY2int2-N *in 'Yali'). As described above, relationships revealed by *Adh1-1c *were poorly resolved, and most intra-species and intra-individual sequences were polymorphic. As shown in Figure 3, intra-individual sequences of *P. calleryana*, *P. hondoensis *and *P. dimorphophylla *were polyphyletic, and the occidental species were not monophyletic. However, the putative interspecific hybrids, *P. × hopeinensis *and *P. × phaeocarpa*, were monophyletic, which was incongruent with other gene trees and our previous understanding of these species. In contrast, only four accessions were polymorphic in the *LFY2int2-N *tree, including *P. × phaeocarpa*, *P. × hopeiensis*, and 'Korlaxiangli' (*P. × sinkiangensis*) (marked in red in Figure 6), all of which were putative interspecific hybrids. Therefore, lineage sorting of ancestral polymorphic *Adh1-1c *alleles may have occurred during diversification of *Pyrus*.

The phylogenetic relationships revealed by *LFY2int2-N *were mostly congruent to other orthologous gene trees and previous studies based on other data. Close relationships among *P. calleryana*, *P. dimorphophylla*, *P. pashia*, *P. pyrifolia*, and/or *P. × serrulata *were supported by all gene trees, suggesting a close relationship among these species. Two distinct *LFY2int2-N *sequences of 'Korlaxiangli' (*P. sinkiangensis*) were grouped with occidental species and *P. pyrifolia*, respectively (Figure 6). Similar relationships were found in *Adh1-1a*, *Adh1-1c *and *Adh1-2 *clades (Figure 3), which supports the hypothesis that *P. sinkiangensis *is an interspecific hybrid involving at least *P. communis *and *P. pyrfolia *[11]. Intra-individual copies of *P. × hopeiensis *and *P*. × *phaeocarpa *were grouped with *P. ussuriensis*, *P. hondoensis *or *P. betulaefolia *in both the *Adh1-1a *clade (Figure 3) and *LFY2int2-N *(Figure 6) clade. *Pyrus *× *phaeocarpa *was a putative hybrid involving *P. betulaefolia *and *P. ussuriensis*, and *P. × hopeiensis *was a hybrid involving *P*. × *phaeocarpa *and *P. ussuriensis*. *Pyrus hondoensis*, which was once classified as a variety of *P. ussuriensis *by morphological data [3], and *P. ussuriensis *were found to be closely related [12]. Phylogenetic relationships among these species were supported by multiple orthologous gene data, suggesting ancient hybridization rather than lineage sorting. More wild individuals of these species are needed to test such complex evolutionary histories.

The relationships based on all separate orthologs were mostly poorly resolved. In our study, different *Adh *and *LEAFY *paralogs showed a relative low sequence divergence (< 0.03). *LFY2int2-N *showed the highest proportion of informative sites (38/562, 6.8%, Table 4) which was similar to results in *Neillia *and *Stephanandra *(7.4%) [39]. Low sequence divergence of multiple DNA regions suggests rapid radiation during divergence [77] and this has been hypothesized for many Pyreae taxa (the former Maloideae taxa) [26,40]. This may also explain the poor resolution of the gene trees. Another contribution to the poor resolution in this study is the conflicting signals caused by recombinants. Recombinants are derived from two homologous chromosomes in one genome during meiosis (genic recombinants) or PCR (artifacts), leading to incorrect phylogenetic inferences [78-80]. As predicted by statistical principles, we found that putative recombinants formed separated clades. Recombinants represent substitutions of two distinct lineages, and thus receive no bootstrap support from either of the lineages in a cladistic phylogeny (data not shown). In this study, most putative recombinants represent one of the intra-individual polymorphic copies and were excluded from analyses. The four *LFY2int2-N *copies of *P. xerophila *all displayed the characteristics of recombinants, and formed separate clades in the tree (Figure 6). Polymorphic *LFY2int2-N *copies in *P. xerophila *may all be ancient genetic recombinants that arose by interspecific hybridization involving both oriental and occidental species. More individuals of *P. xerophila *and occidental species are necessary to confirm this hypothesis and investigate the origin of this species.

### Phylogenetic utility of the introns

Among three *Adh1-1 *subparalogs, *Adh1-1c *was orthologous and recovered in all accessions, but it resulted in a poorly resolved phylogeny due to lack of informative sites and possible lineage sorting (Table 3). This makes it inadequate for the phylogenic reconstruction of *Pyrus*. The two introns of *Adh1-2*, *Adh2-1 *and *Adh2-2*, were not sequenced and analyzed in the current study, and it is unknown whether multiple subparalogs also exist for these paralogs. *Adh2-2 *might not exist in *Pyrus *species as discussed above. The phylogenetic utility of the introns of *Adh2-1 *and *Adh1-2 *needs to be estimated, which will require primers designed specifically to the paralogs.

*LFY1int2 *was not suitable for studying interspecific relationships due to sorting of *LFY1int2-a *and *LFY1int2-b *paralogs and the unclear origin of *LFY1int2-c*. In contrast, *LFY2int2-N *showed the highest sequence divergence, resulting in the best-resolved tree. Inparalogs of *LFY2int2-Ins8 *and *LFY2int2-Del2*, as well as the *LFY2int2-S *of unclear origin, could be easily identified and removed from phylogenetic inferences. Most importantly, relationships based on *LFY2int2-N *were congruent to previous studies based on morphological and molecular marker data. Conflicting placement of species may be resolved by using *LFY2int2-N*. It provides reliable evidence of ancient hybridization, since incomplete lineage sorting was not imputed for *LFY2int2-N*. Phylogenetic studies of *Pyrus *based on nuclear gene regions have been rare. Only the ITS region has been applied to a wide range of East Asian *Pyrus *species, but it resulted in a poorly resolved tree [28]. One study based on the 18S gene focused only on two species, *P. pyrifolia *and *P. communis *[81]. We conclude that *LFY2int2-N *is currently the most useful nuclear gene region for phylogenetic inference in *Pyrus*. It is as yet unknown whether additional inparalogs representing *LFY2int2 *will be found by analyzing more occidental species and individuals of oriental species.

## Conclusion

This is the first study that explores LCNGs for phylogenetic analyses in *Pyrus*. It is also the first to document the gene structures and transcription of *Adh *homologs in the Rosaceae taxa. We demonstrated that frequent gene duplications contributed to complex outparalogs and inparalogs of *Adh *genes with functional diversification or nonfunctionalization. Paralogs, lineage sorting of alleles, and recombinants are three major problems when applying LCNGs in plant phylogenetic analyses. One ortholog of *LEAFY*, *LFY*2*int2-N*, is currently the best nuclear marker for studying interspecific relationships of *Pyrus*. Complex reticulate histories likely complicate the phylogenetic reconstruction of some *Pyrus *species. To better resolve interspecific relationships and examine the evolutionary processes of *Pyrus*, we are extending our phylogenetic studies with plastid DNA and nuclear DNA, including *LFYint2-N*, and by sampling a wider assortment of species and individuals.

## Methods

### Taxon sampling, DNA extraction, primer design and amplification

Twenty-five accessions from 13 oriental species and four occidental species of genus *Pyrus *were included. Six accessions of four *Malus *species were used as outgroups (Table 1). Total genomic DNA was isolated from fresh leaf tissue using a modified sodium dodecyl sulfate (SDS) method [12,82].

To date, a complete cDNA sequence representing *Adh1 *from 'Granny Smith' (*M. domestica*, Z48234) and two 5' partial (beginning at the 3' end of exon 2) cDNA sequences representing two distinct *Adh *loci (AF031900-*Adh1*, AF031899-*Adh2*) from 'Packham's Triumph' (*P. communis*) are available. The *Adh *series (*Adh1 *and *Adh2*) were named randomly and do not correspond to previously named alleles. A forward primer (Adh-F1) based on sequence of Z48243 and three downstream primers (Adh1-R1, Adh1-R2 and Adh2-R) based on AF031900-*Adh1 *and AF031899-*Adh2 *were designed to obtain the entire gene region in several accessions including *P. communis*, 'Flemish Beauty' (*P. communis*), 'Nanguoli' (*P. ussuriensis*), 'Cuiguan' (*P. pyrifolia*), 'Korlaxiangli' (*P. sinkiangensis*), 'Ralls' (*M. domestica*), *M. rockii *and *M. domestica *subsp. *chinensis*. However, these primer pairs only succeeded in amplifying 12 *Adh1 *sequences in select accessions. Therefore, two additional forward primers, Adh1-F2 and Adh2-F, were designed based on AF031900-*Adh1 *and AF031899-*Adh2*, respectively, targeting a partial *Adh *region lacking exon 1 and intron 1. These primers amplified *Adh2 *sequences and additional *Adh1 *sequences. All of the above PCR products were designated as long partial genomic *Adh *sequences (G-PCR). Considering labor costs and difficulties in amplifying and sequencing fragments greater than 2 kb, a smaller region covering only introns 2 and 3 (about 650 bp) of *Adh1 *(reduced *Adh1*) was used in all accessions to construct a phylogeny.

For *LEAFY*, a long partial region of *LFY1 *and *LFY2 *spanning exon 2 and intron 2 was first amplified in some accessions using the primer pair 'LFY-F+LFY-R' developed in an exonic region of *M. domestica *'Pinova' (DQ535885, DQ535886). After initial sequence analyses, specific primer pairs of 'LFY1-F + LFY1-R' and 'LFY2-F + LFY2-R' were developed to amplify partial intron 2 of *LFY1 *and *LFY2 *(*LFY1int2*, *LFY2int2*), respectively. This was done independently in all accessions except the three commercial cultivars ('Cuiguan', 'Ralls', 'Flemish Beauty' and 'Fuji'). Sequence information for primers used in this study is listed in Table 2 and their locations illustrated in Figure 1.

PCR was carried out in a final reaction volume of 50 *μ*L, containing 10-20 ng total DNA, 2.5 mM MgCl_2_, 0.4 *μ*M of each primer, 5% DMSO (v/v), 0.2 mM dNTP, 2 U *Taq *DNA polymerase (Takara Biotechnology Company Co., Ltd, Kyoto, Japan) and 1 × PCR buffer supplied by the manufacturer. Amplification of the long partial *Adh *region was performed for 4 min at 94°C, followed by 35 cycles of 40 s at 94°C, 40 s at 58°C, 2 min of 20 s at 72°C, and a final extension for 7 min at 72°C. For other shorter regions like locus-specific RT-PCR as described below, the PCR procedure was identical, but only 1 min was needed for the extension step.

### Cloning and sequencing

PCR products were verified by 2% agarose gel electrophoresis, and the target bands were separated and purified using 3S spin DNA Agarose Gel Purification (Shenergy Biocolor, Shanghai, China). For long partial genomic *Adh *amplified by G-PCR, the purified PCR products were cloned using TA cloning kit Pmd19 (Takara) and more than three clones per sample were sequenced using M13^+^, M13^- ^primers and internal primers located at exonic regions (Adh1-F5 for *Adh1*, and Adh2-F5 for *Adh2*, Table 2). For the reduced *Adh1 *region, five to ten clones per sample were sequenced using the M13^+ ^primer. For the long partial *LEAFY *region, more than three clones were sequenced to obtain reads representing putative *LFY1 *and *LFY2*. For *LFY1int2 *and *LFY2int2*, the purified PCR products were directly sequenced by amplification primers. Additionally, more than three clones were sequenced to capture all the copies indicated by direct sequencing results.

### Sequence analyses

Intronic and exonic boundaries were determined by comparison with available cDNA sequences and preservations of the 'GT' and 'AG' at two ends of introns. Sequences were aligned with Clustal X [83]. Sequence divergence within and between different homologs was calculated using MEGA4 [84] with gaps treated as pairwise deletions. Putative recombinants were detected using RDP3 software package [54], and some putative recombinants were identified manually.

### Transcription of Adh homologs

The expression patterns of *LFY1 *and *LFY2 *have been well documented in *Malus *and *Pyrus *[41], but little was known about expression patterns of *Adh1 *and *Adh2 *in *Pyrus*. Therefore, 'Cuiguan' (*P. pyrifolia*), 'Nanguoli' (*P. ussuriensis*), 'Korlaxiangli' (*P. sinkiangensis*) and 'Flemish Beauty' (*P. communis*), representing four major pear cultivar groups together with 'Fuji' (*M. domestica*) were selected to examine transcription of *Adh1 *and *Adh2*. For 'Cuiguan' (*P. pyrifolia*), fresh young leaves, ripe fruits and seeds were collected in our campus yard for *Adh *expression analyses, while for the other accessions, only ripe fruits were used. The plant tissues were frozen in liquid nitrogen and stored at -80°C. Total RNA was isolated using a modified CTAB method. First strand cDNA was synthesized from 1.0 μg of total RNA using a poly (T)_18 _as primer and AMV reverse transcriptase (Bio Basic Inc, New York, USA) according to the manufacturer's instructions. To efficiently detect transcription of *Adh1 *and *Adh*2 independently, two specific primer pairs 'spAdh1-F+spAdh1-R' and 'spAdh2-F+spAdh2-R' (Table 2) targeting a shorter region were used for RT-PCR and specific genomic PCR (SG-PCR). PCR products were directly sequenced followed by cloning to identify copies involved in transcription. *Actin *was analyzed as a reference gene. The primers Pact-F and Pact-R were designed based on the *Actin *gene sequences from *P. communis *and 'Yali' (*P. pyrifolia*, CWP) (AB190176, GU830958) (Table 2).

### Phylogenetic analyses

The predicted amino acid sequences of long partial *Adh *genes in *Pyrus *and *Malus *from G-PCR were compared with those from other well-studied plant taxa by conducting NJ and MP analyses using PAUP 4.0b10 [85]. For reduced *Adh1*, nucleotide sequences including the exonic and intronic regions were both included. *LFY1int2 *and *LFY2int2 *were analyzed separately, since their sequence homology was too low to be aligned. MP analyses were conducted using PAUP 4.0b10 with gaps treated as missing data. MP analyses were performed using a heuristic search with the TBR and Multree options. To estimate support for the clades, non-parametric bootstraps were estimated with 1000 replicates. Bayesian analyses were performed with MrBayes 3.1 [86]. The best fitting substitution models for each dataset were determined with the Akaike Information Criterion (AIC) using ModelTest 3.06 [87]. The AIC favored the HKY+G for the reduced *Adh1 *and the K81uf+Gfor both *LFY1int2 *and *LFY2int2*. Markov chains were run for 10,000,000 generations with sample frequency of 100. The average standard deviation of split frequency was 0.003 for reduced *Adh1*, 0.005 for *LFY1int2 *and 0.002 for *LFY2int2*, indicating the runs have reached convergence for each dataset. The first 25% of the trees were discarded as burn-in. Clade posterior probabilities were calculated from the combined sets of trees. Both MP and Bayesian analyses resulted in largely congruent tree topologies. Sequences included in the final phylogenetic analyses were deposited in GenBank (Accessions GU991401-991522, HM003976-004066, HQ912028-HQ912076). Alignments of these datasets are deposited as additional files 4, 5, 6 and 7.

## Authors' contributions

XZ carried out primer design, molecular phylogenetic analyses, and drafted the manuscript. CH carried out genomic DNA and RNA isolation, participated in the amplifications and cloning. DS helped in writing the manuscript. JL participated in the amplification and collection of fresh leaves. JC participated in the revision. YT conceived of the study and participated in the revision. All authors read and approved the final manuscript.

## Supplementary Material

Additional file 1 **50% majority-rule consensus tree based on amino acid sequences of *Adh *loci from diverse plant taxa**. ADH sequences in Rosaceae are highlighted in blue. Numbers above the branches or near the branch nodes indicate bootstrap values (1000 replicates). Accession number was given for sequences from GenBank. Multiple intraindividual sequences for *Adh1 *(*Adh1-1 *and *Adh1-2*) and *Adh2 *(*Adh2-1 *and *Adh2-2*) are differentiated by the number in the brackets following the taxa name*: Though *Adh2-1 *was not obtained by G-PCR in *Malus*, its transcription was detected by RT-PCR in 'Fuji' (*M. domestica*), which was described in the text. Click here for file

Additional file 2 **Transcription of *Adh1 *homologs revealed by neighbor joining (NJ) analyses**. Sequences obtained from genomic PCR are marked by G followed by the corresponding *Adh1-1 *subparalogs name in the square brackets. Sequences obtained from RT-PCR are marked by RT followed by the plant tissues used in parenthesis. Sequences obtained from specific genomic PCR are marked by SG. Multiple intraindividual sequences obtained from different PCR or plant tissues are differentiated by the fraction in the brackets following the taxa name. Putative pseudogenes obtained by G-PCR are marked by '**ψ**'. Click here for file

Additional file 3 **Transcription of *Adh2 *homologs revealed by neighbor joining (NJ) analyses**. Sequences obtained from genomic PCR are marked by G followed by the paralogs name in the square brackets. Sequences obtained from RT-PCR are marked by RT followed by the plant tissues used in parenthesis. Sequences obtained from specific genomic PCR are marked by SG. Multiple intraindividual sequences obtained from different PCR or plant tissues are differentiated by the fraction in the brackets following the taxa name. Putative pseudogenes obtained by G-PCR are marked by '**ψ**. Click here for file

Additional file 4 **Alignment of ADH amino acid sequences from different plant taxa**. Click here for file

Additional file 5 **Alignment of the reduced *Adh1 *nucleotide sequences from *Malus *and *Pyrus***. Click here for file

Additional file 6 **Alignment of *LFY1int2 *nucleotide sequences from *Malus *and *Pyrus***. Click here for file

Additional file 7 **Alignment of *LFY2int2 *sequences from different plant taxa**. Click here for file
