# Supplementary figures and images for: Molecular evolution of Adh and LEAFY and the phylogenetic utility of their introns in Pyrus (Rosaceae)
Source: BMC Evol Biol. 2011 Sep 14;11:255. doi: 10.1186/1471-2148-11-255 (PMC3182939; doi:10.1186/1471-2148-11-255)

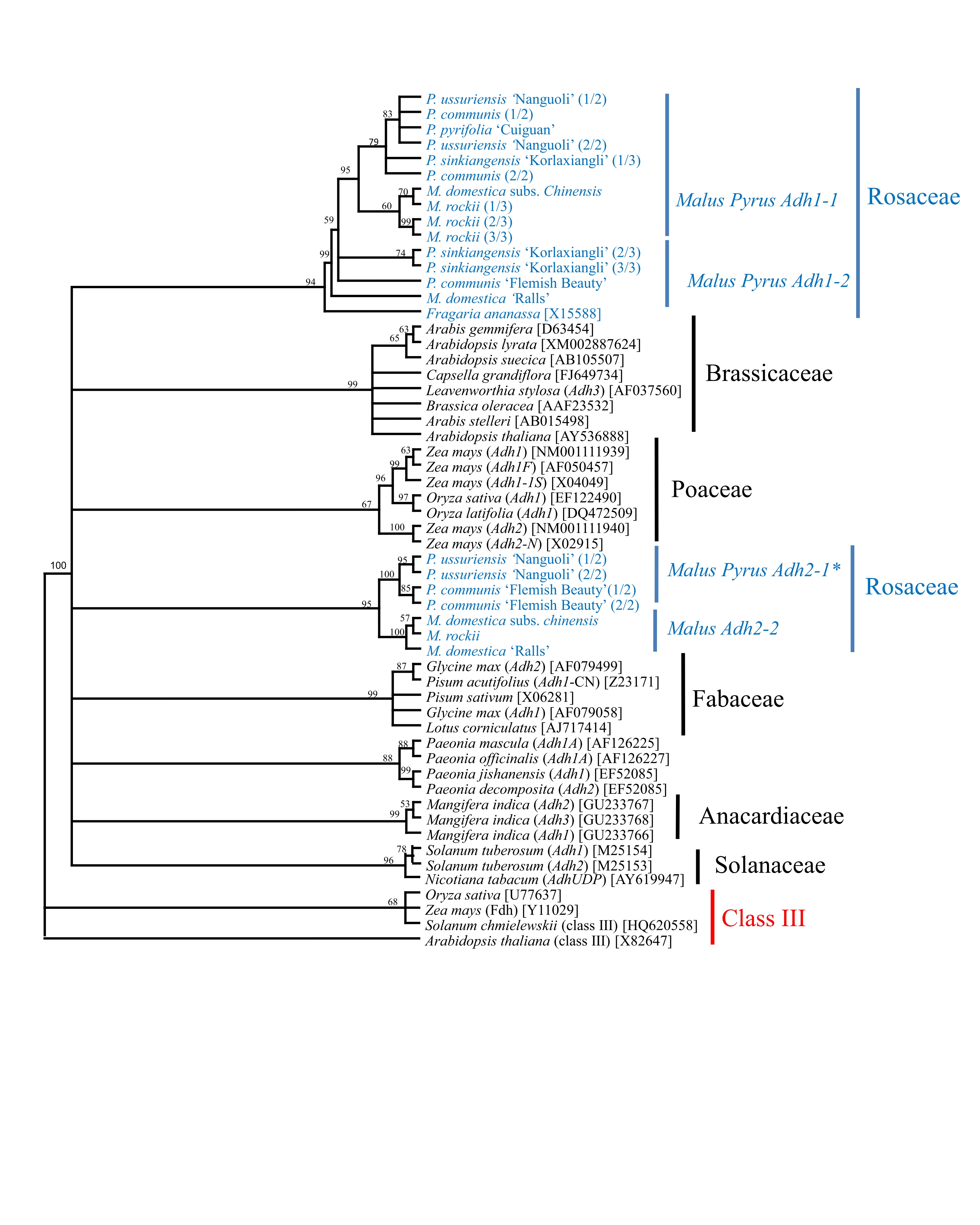

Supplement: Additional file 1 — 50% majority-rule consensus tree based on amino acid sequences of Adh loci from diverse plant taxa. ADH sequences in Rosaceae are highlighted in blue. Numbers above the branches or near the branch nodes indicate bootstrap values (1000 replicates). Accession number was given for sequences from GenBank. Multiple intraindividual sequences for Adh1 (Adh1-1 and Adh1-2) and Adh2 (Adh2-1 and Adh2-2) are differentiated by the number in the brackets following the taxa name*: Though Adh2-1 was not obtained by G-PCR in Malus, its transcription was detected by RT-PCR in 'Fuji' (M. domestica), which was described in the text. [file 1471-2148-11-255-S1.JPEG]

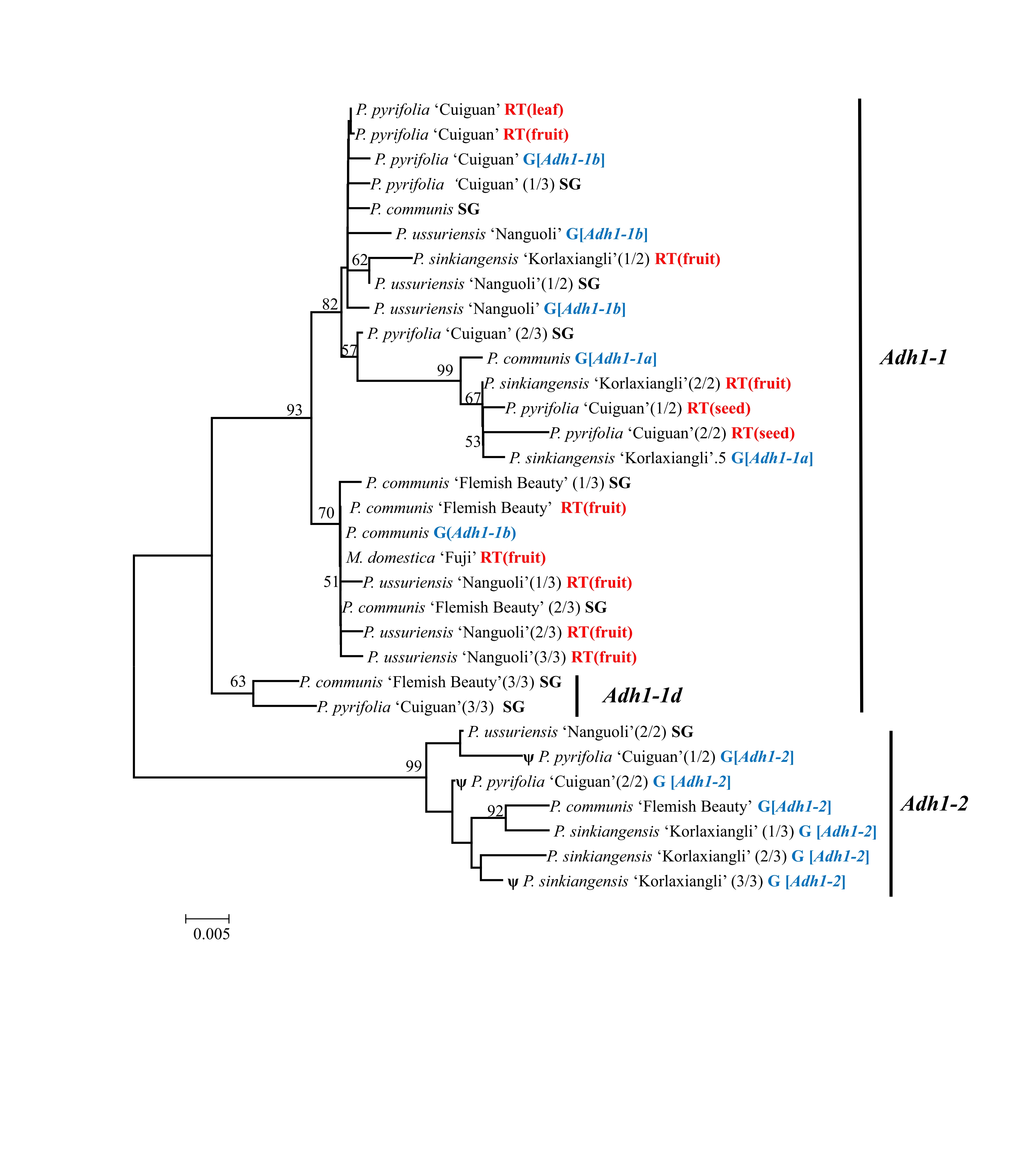

Supplement: Additional file 2 — Transcription of Adh1 homologs revealed by neighbor joining (NJ) analyses. Sequences obtained from genomic PCR are marked by G followed by the corresponding Adh1-1 subparalogs name in the square brackets. Sequences obtained from RT-PCR are marked by RT followed by the plant tissues used in parenthesis. Sequences obtained from specific genomic PCR are marked by SG. Multiple intraindividual sequences obtained from different PCR or plant tissues are differentiated by the fraction in the brackets following the taxa name. Putative pseudogenes obtained by G-PCR are marked by 'ψ'. [file 1471-2148-11-255-S2.JPEG]

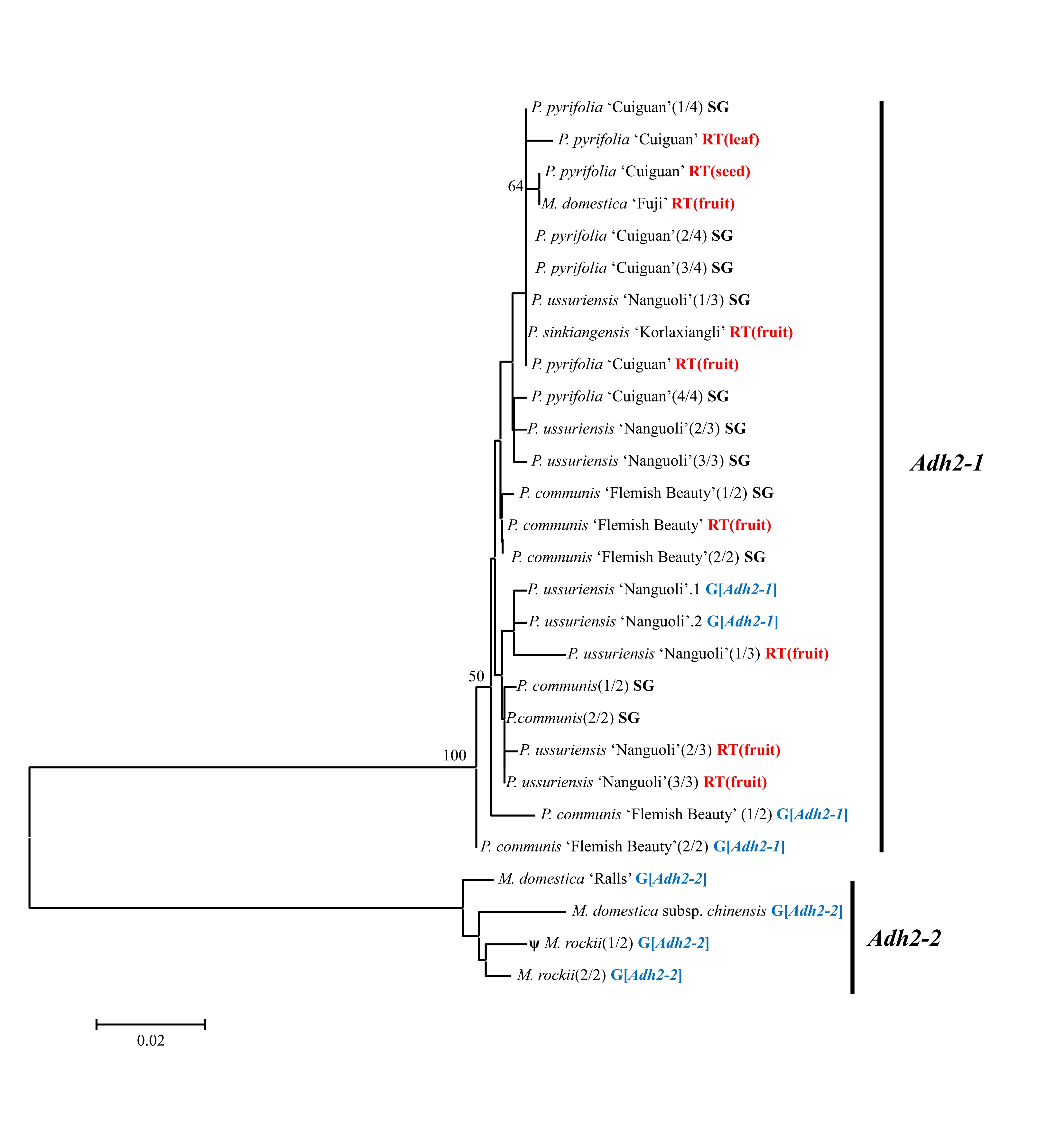

Supplement: Additional file 3 — Transcription of Adh2 homologs revealed by neighbor joining (NJ) analyses. Sequences obtained from genomic PCR are marked by G followed by the paralogs name in the square brackets. Sequences obtained from RT-PCR are marked by RT followed by the plant tissues used in parenthesis. Sequences obtained from specific genomic PCR are marked by SG. Multiple intraindividual sequences obtained from different PCR or plant tissues are differentiated by the fraction in the brackets following the taxa name. Putative pseudogenes obtained by G-PCR are marked by 'ψ. [file 1471-2148-11-255-S3.JPEG]
